# Phase II study of ruxolitinib, a selective JAK1/2 inhibitor, in patients with metastatic triple-negative breast cancer

**DOI:** 10.1038/s41523-018-0060-z

**Published:** 2018-05-04

**Authors:** Daniel G. Stover, Carlos R. Gil Del Alcazar, Jane Brock, Hao Guo, Beth Overmoyer, Justin Balko, Qiong Xu, Aditya Bardia, Sara M. Tolaney, Rebecca Gelman, Maxwell Lloyd, Yu Wang, Yaomin Xu, Franziska Michor, Vivian Wang, Eric P. Winer, Kornelia Polyak, Nancy U. Lin

**Affiliations:** 1 Department of Medical Oncology, Dana-Farber Cancer Institute, Boston, MA USA; 2 Department of Biostatistics & Computational Biology, Dana-Farber Cancer Institute, Boston, MA USA; 3 Department of Cancer Biology, Vanderbilt University Medical Center, Nashville, TN USA; 4 Department of Biostatistics, Vanderbilt University Medical Center, Nashville, TN USA; 5 Department of Medical Oncology, Massachusetts General Hospital, Boston, MA USA; 6 Department of Biostatistics, Harvard T. H. Chan School of Public Health, Boston, MA USA; 7 Department of Stem Cell and Regenerative Biology, Harvard University, Cambridge, MA USA; 8 Center for Cancer Evolution, Dana-Farber Cancer Institute, Boston, MA USA; 9 Present Address: Department of Medical Oncology, Ohio State University Comprehensive Cancer Center, Columbus, OH USA

## Abstract

Preclinical data support a role for the IL-6/JAK2/STAT3 signaling pathway in breast cancer. Ruxolitinib is an orally bioavailable receptor tyrosine inhibitor targeting JAK1 and JAK2. We evaluated the safety and efficacy of ruxolitinib in patients with metastatic breast cancer. This was a non-randomized phase II study enrolling patients with refractory, metastatic triple-negative breast cancer. The primary endpoint was objective response by RECIST 1.1. The study was designed to enroll patients whose archival tumor tissue was pSTAT3-positive (*T*-score >5) by central immunohistochemistry. pSTAT3 staining was available from 171 of 217 consented patients and pSTAT3 *T*-score was positive in 67/171 (39.2%) tumors, suggesting that JAK–STAT activation is frequent. Twenty-three patients including one patient with inflammatory breast cancer were enrolled. Ruxolitinib was well-tolerated with infrequent grade 3 or higher toxicities with fatigue as the most common toxicity. Among 21 patients who received at least one dose of protocol therapy, no objective responses were observed and the study was closed to further accrual. Pharmacodynamic analyses of baseline vs. cycle 2 biopsies suggest on-target activity, including a significant decrease in the proportion of pSTAT3^+^ cells in three patients with paired biopsies and downregulation of JAK–STAT target genes and signatures via transcriptional analyses of 11 total baseline and four metastatic biopsies. Immuno-FISH analyses demonstrate intratumoral heterogeneity of pSTAT3 and *JAK2* amplification. Ruxolitinib, as a single agent, did not meet the primary efficacy endpoint in this refractory patient population despite evidence of on-target activity.

## Clinical trial: JAK inhibitor not effective for triple-negative breast cancer

A drug that blocks the Janus tyrosine kinase (JAK) pathway offered no clinical benefit to women with triple-negative breast cancer. Ruxolitinib is an inhibitor of JAK1 and JAK2 that is approved to treat certain rare cancers of the blood and bone marrow, and there’s a good scientific evidence to think it might have anti-tumor effects against breast cancer as well. So a team led by N.U.L. from the Dana-Farber Cancer Institute in Boston, Massachusetts, USA, conducted a small phase II trial to evaluate ruxolitinib in women with refractory, metastatic, triple-negative disease whose tumors tested positive for high expression of pSTAT3, a protein in the same signaling pathway as JAK. Yet, despite the fact that pSTAT3 levels went down among the 21 women who received the drug, not a single one responded to the therapy.

## Introduction

Triple-negative breast cancer (TNBC) is defined by the absence of expression of the estrogen receptor, progesterone receptor, and HER2 amplification and accounts for 12–17% of all breast cancers.^1,2^ TNBCs are associated with aggressive features and poor outcomes, particularly in the metastatic setting, in which median survival is only 15–21 months.^3–5^ The likelihood of an objective response or prolonged clinical benefit to chemotherapy in the late-stage setting is low.^4,6^ To date, no targeted agents have been approved for TNBC.

CD44^+^CD24^–^ tumor cells have stem-like characteristics and are thought to contribute to metastatic progression and therapeutic resistance.^7,8^ They are present in nearly 100% of basal-like tumors and appear to be enriched in residual disease after neoadjuvant chemotherapy.^9,10^ Through an shRNA screen for genes required in CD44^+^CD24^–^ breast cancer cells, the IL-6/JAK2/STAT3 pathway was identified as a key driver of the phenotype.^11^ Treatment with a pan-JAK inhibitor preferentially decreased the viability of basal-like cell lines and in preclinical xenografts of both basal-like cell lines and primary breast cancer cells, JAK2 inhibitors led to reduced tumor size.^11^ In addition, *JAK2* amplification is present in approximately 11% of primary TNBCs^12^ and enriched in residual disease after neoadjuvant chemotherapy.^13^ Furthermore, work from several groups has implicated the JAK2/STAT3 pathway in the pathogenesis of inflammatory breast cancer.^14,15^

Ruxolitinib is an orally bioavailable receptor tyrosine kinase inhibitor, which targets JAK1 and JAK2. It is approved for the treatment of patients with intermediate or high-risk myelofibrosis and those with polycythemia vera who have had an inadequate response to or are intolerant of hydroxyurea.^16,17^ The most commonly observed toxicities are anemia, leukopenia, thrombocytopenia, bruising, dizziness, and headache. We hypothesized that ruxolitinib would have anti-tumor efficacy in breast cancer. To test this hypothesis, we conducted a non-randomized phase II study to evaluate its safety and efficacy in patients with refractory, metastatic TNBC in patients whose archival tumor tissue was pSTAT3-positive (*T*-score >5) by central immunohistochemistry. We performed detailed correlative studies including analyses of ruxolitinib pharmacodynamics, JAK–STAT signaling, and immunophenotyping.

## Results

### Patients and treatment

**Fig. 1:**
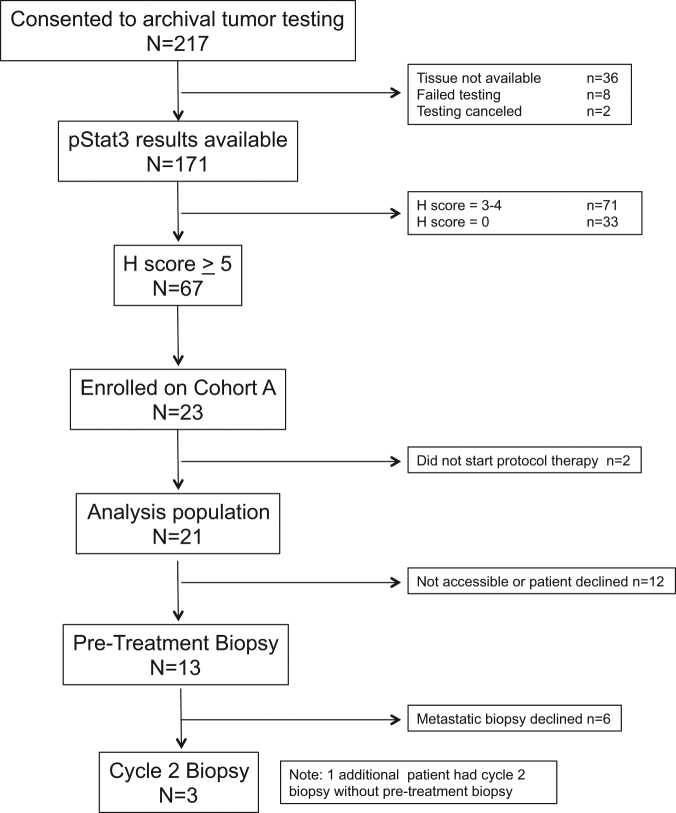
CONSORT diagram

A total of 217 patients consented to archival primary tumor testing (“pre-screening”; Fig. 1). Results of pSTAT3 testing were available for 171. Of these, *T*-score was >5 in 67 patients; 3–4 in 71 patients, and 0 in 33 patients. Patients with a *T*-score of >5 during “pre-screening” could subsequently elect to consent to the therapeutic portion of the trial at the time of disease progression. Between October 2012 and June 2014, 23 patients were enrolled into Cohort A, of whom 21 patients received at least one dose of protocol therapy and are included in the safety and efficacy analyses. Follow-up information is available through 1 June 2015. At the time of data cutoff, all patients had discontinued protocol therapy and 17 (81%) had died. The reasons for discontinuation of protocol therapy were progressive disease by RECIST 1.1 (*n* = 10, 48%), progressive disease by clinical assessment (*n* = 9, 43%), unacceptable toxicity (*n* = 1, 5%), or both toxicity and clinical progression (*n* = 1, 5%). Patient and treatment characteristics are shown in Table 1. Patients were heavily pre-treated, with nearly half (48%) having received three or more prior lines of chemotherapy in the metastatic setting.

**Table 1:** Baseline patient, tumor, and treatment characteristics

Characteristics	All (*n* = 21)
	No. of patients (%)
Age, years	
Median (range)	51 (36–72)
Race	
White	18 (86%)
Black or African-American	1 (5%)
Asian	1 (5%)
Other	1 (5%)
ECOG PS at baseline	
0	9 (43%)
1	11 (52%)
2	1 (5%)
Disease-free interval	
<2 years	16 (76%)
>2 years	4 (19%)
Stage IV disease at diagnosis	1 (5%)
Inflammatory breast cancer^a^	1 (5%)
Adjuvant or neoadjuvant chemotherapy	
Adjuvant or neoadjuvant anthracycline	17 (81%)
Adjuvant or neoadjuvant taxane	16 (76%)
Lines of chemotherapy for metastasis or recurrence	
Median (range)	2 (1–8)
None	0 (0%)
1 line	6 (29%)
2 lines	5 (24%)
3+ lines	10 (48%)
Prior chemotherapy for metastasis or recurrence	
Capecitabine	9 (43%)
Platinum salt	12 (57%)
Eribulin	5 (24%)

^a^The single patient with inflammatory breast cancer enrolled had ER-negative, PR-negative, and HER2-negative disease

### Safety

A total of 40 cycles of ruxolitinib was administered. The median number of cycles per patient was 2 (range 1–5). Dose holds occurred in six (28%) patients. Only one patient required a dose reduction (for neutropenia) and ultimately discontinued therapy for toxicity. Hematologic toxicity was consistent with the known adverse effect profile of the drug (Table 2, Supplemental Table S1). Anemia was observed in six (28%) patients, including three patients with grade 3 anemia. Neutropenia was observed in three patients; however, there were no cases of febrile neutropenia. Thrombocytopenia was observed in three (14%) patients (none grade 3 or higher). Fatigue was the most commonly reported non-hematologic event. Other reported adverse events included dyspnea, anorexia, and cough, though these were primarily attributed to patients’ underlying disease process. Between baseline and cycle 2, there were no statistically significant differences in patient-reported global health status score, functional scales, and symptom scales as assessed by the European Organization for Research and Treatment of Cancer (EORTC) QLQ-C30 or the MD Anderson Symptom Inventory (MDASI) mean core scores or mean interference scores.

**Table 2:** Summary of events with at least 10% incidence or grade 3 or 4—all relatedness

	Grade				Total *N* (%)
Toxicity description	Mild	Moderate	Severe	Life-threatening	
Alanine aminotransferase increased	1	2	0	0	3 (14)
Alkaline phosphatase increased	1	2	1	0	4 (19)
Anemia	1	2	3	0	6 (28)
Anorexia	2	3	0	0	5 (24)
Ascites	0	0	1	0	1 (5)
Aspartate aminotransferase increased	1	1	2	0	4 (19)
Constipation	2	1	0	0	3 (14)
Cough	4	1	0	0	5 (24)
Dyspnea	4	2	0	0	6 (28)
Edema limbs	1	2	0	0	3 (14)
Fatigue	8	4	0	0	12 (57)
GGT increased	0	0	1	0	1 (5)
Hyponatremia	0	0	1	0	1 (5)
Nausea	2	1	0	0	3 (14)
Neutrophil count decreased	0	1	2	0	3 (14)
Pain	0	4	0	0	4 (19)
Peripheral sensory neuropathy	3	0	0	0	3 (14)
Platelet count decreased	2	1	0	0	3 (14)
Pleural effusion	0	3	0	0	3 (14)
Syncope	0	0	1	0	1 (5)
Vascular disorders—other	0	0	0	1	1 (5)
White blood cell decreased	0	0	1	0	1 (5)

### Efficacy

Twenty-one patients received at least one dose of study drug and are included in the efficacy analysis. No patient achieved a complete or partial response. Three (14%) patients experienced stable disease, but none for more than 24 weeks. For 17 patients, best response was progressive disease by RECIST 1.1. One patient was unevaluable for response due to treatment discontinuation for toxicity prior to the first restaging evaluation. Median progression-free survival (PFS) was 1.2 months (95% CI 0.97–1.84). Median overall survival (OS) was 4.5 months (95% CI 2.9–10.2; Supplemental Fig. S1).

### JAK/STAT pathway and drug effect correlative analyses

**Fig. 2:**
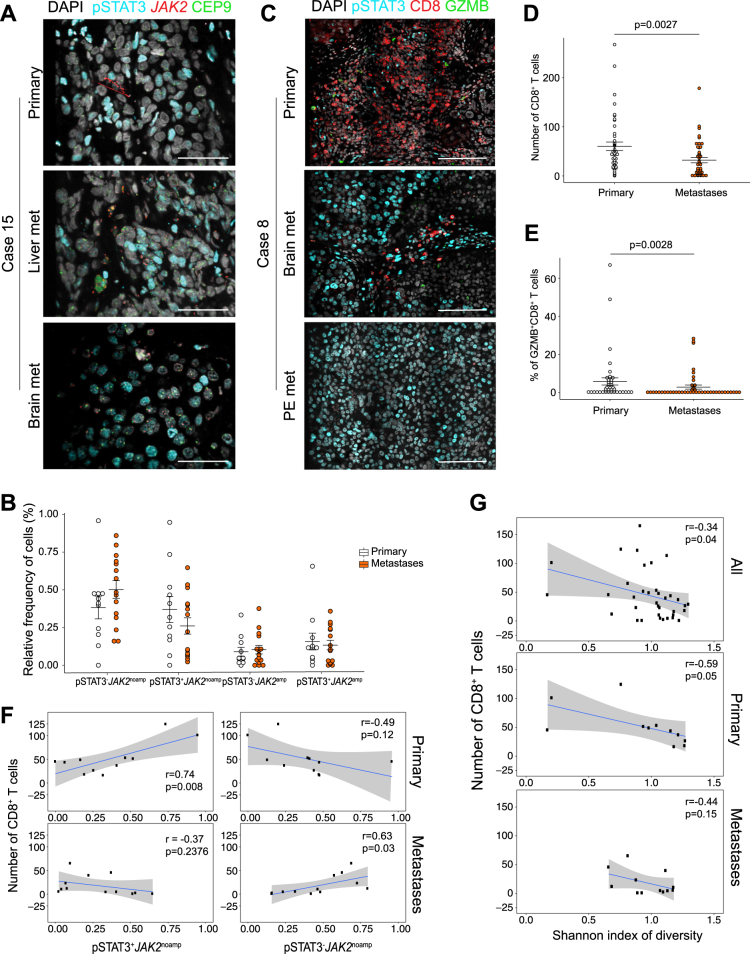
Immuno-FISH and CD8+ T-cell infiltrate in primary vs. metastatic samples. **a** iFISH images of one area of the primary tumor and brain and liver metastases of case 15. Scale bar 100  mm. **b** Dot plot depicting relative frequencies of the four different cell types in primary and metastatic samples. Error bars, S.E.M. **c** Immunofluorescence analysis of CD8, GranzymeB (GZMB), and pSTAT3. Images are a montage of nine fields captured from one area of the tissue. Scale bar 100  mm. **d**, **e** Graph depicting numbers of CD8^+^ T cells per montage (**d**) and fraction of GZMB^+^CD8^+^ T cells (**e**). Significance of the difference between primary and metastatic samples was calculated using the Wilcoxon rank-sum test. **f** Correlation analysis between the number of infiltrating CD8^+^ T cells and the relative frequencies of specific cell populations in primary and metastatic samples. **g** Correlation analysis between number of infiltrating CD8^+^ T cells and the Shannon index of diversity in all, primary and metastatic samples. Gray area, 95% confidence interval. Sample sizes were *n*  =  16 primary tumors and *n*  =  18 metastases

To investigate on-target activity of ruxolitinib, we investigated tissue-based and transcriptional evidence of JAK–STAT signaling inhibition comparing metastatic biopsies obtained pre-treatment and prior to cycle 2. A total of 14 patients underwent study biopsy with nine patients having baseline biopsy only, one patient having cycle 2 biopsy only, and three patients with both baseline and cycle 2 biopsies. Eleven of 13 baseline and all 4 metastatic biopsies passed tumor content quality control and were included in transcriptional analyses. In three patients with paired biopsies, there was a significant reduction in the fraction of pSTAT3^+^ cells after one cycle of treatment (mean pSTAT3^+^ 64.8% pre-treatment vs. 34.7% prior to cycle 2, *p* = 0.029; Fig. 2a). In addition, we evaluated whole transcriptome RNA sequencing from biopsies obtained pre-treatment (*n* = 14) and prior to cycle 2 (*n* = 4). JAK/STAT-induced target genes *SOCS3* and *EGFR* were significantly repressed with ruxolitinib treatment (Student’s *t*-test *p* = 0.004 and *p* = 0.036, respectively) while JAK/STAT-repressed gene *LCK* was significantly upregulated (*p* = 0.007; Fig. 2b). As a more robust evaluation of pathway activity, we investigated two independently derived STAT3 signatures,^18,19^ both of which were decreased in cycle 2 biopsy samples (*p* = 0.041 and *p* = 0.065, respectively; Fig. 2c). As an unbiased approach, we identified differentially expressed genes using DESeq2^20^ performed pathway analysis of 44 genes with *p* < 0.001. The top two pathways were “Immune Response/OncostatinM signaling via JAK–STAT” in mouse and human cells (Fig. 2d). As an alternative pharmacocynamic measure, we also explored trends in interleukin-6 (IL-6) and C-reactive protein (CRP) levels at baseline, cycle 2, and end of treatment; however, there were no convincing trends, though the power to detect differences was limited due to small patient numbers (Supplemental Table S2).

**Fig. 3:**
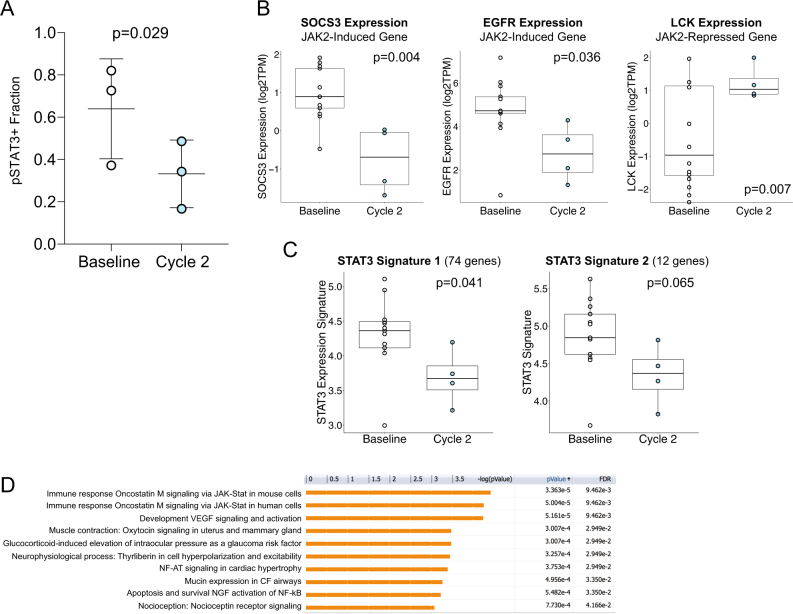
Pre-treatment and cycle 2 biopsies reveal on-targets effect of JAK/STAT pathway suppression. **a** Fraction of cells positive for pSTAT3 in three patients who underwent both pre-treatment and cycle 2 biopsies. **b–d** Total RNA was isolated from fresh frozen biopsy samples obtained at baseline (*n*  =  12) and cycle 2 (*n*  =  4) and RNA sequencing performed. Individual JAK/STAT target genes—including both upregulated (*SOCS3, EGFR*) and downregulated (*LCK*)—demonstrate expression changes in cycle 2 concordant with pathway suppression (**b**). Two independently derived STAT3 pathway gene expression signatures demonstrated suppression at cycle 2 (**c**). GeneGO analyses of differentially expressed genes (DESeq2^20^ *p* <  0.001) were identified revealed that Jak–STAT pathways as the top two ontologies (**d**)

To assess *JAK2* amplification and pSTAT3 status and impact of treatment at the single-cell level, we developed an immunofluorescence-fluorescent in situ hybridization (iFISH) protocol (Fig. 3a). Cells were classified into four distinct populations based on positivity for pSTAT3 (i.e., pSTAT3^+^ and pSTAT3^–^) and JAK2 amplification (i.e., *JAK2*^amp^ and *JAK2*^noamp^; Fig. 3b, Supplemental Fig. S2A). The majority of pSTAT3^+^ cells were JAK2^noamp^, although *JAK2*-amplified cells were more likely to be pSTAT3^+^ (Fig. 3c). There was a decrease in pSTAT3^+^ tumor cells (*JAK2* amplified and non-amplified) in biopsies after two cycles of ruxolitinib treatment, confirming on-target activity of the drug even in the presence of *JAK2* amplification (Fig. 3c, d). We calculated Shannon diversity index of intratumor cellular diversity, a quantitative measure that reflects the number and amount of distinct phenotypes within a tumor, and detected a dramatic decrease in diversity in one patient and a less significant decrease in the other two (Fig. 3e). Collectively, these data demonstrate both tissue-based and transcriptional evidence of JAK–STAT pathway inhibition.

### Association of tumor cell phospho-STAT3 and JAK2 amplification and CD8+ T-cell infiltrate in primary and metastatic TNBCs

We hypothesize resistance to ruxolitinib could be mediated through either intratumoral heterogeneity, given known extensive intratumoral heterogeneity in TNBC,^5,6^ or immune escape, given the importance of JAK2–STAT3 signaling in normal immune cells. Because it remains unclear how intratumoral heterogeneity and immune infiltrates differ between primary and metastatic TNBC tumors, we evaluated paired primary and metastatic lesions from 16 patients with 18 total metastases evaluated. To assess *JAK2* amplification and pSTAT3 status and impact of treatment at the single-cell level, we developed an iFISH protocol (Fig. 2a). Cells were classified into four distinct populations based on positivity for pSTAT3 (i.e., pSTAT3^+^ and pSTAT3^–^) and JAK2 amplification (i.e., *JAK2*^amp^ and *JAK2*^noamp^ (Fig. 2a). We first investigated heterogeneity defined through the relative frequencies of the four cell subpopulations via iFISH between primary tumors and metastatic tumors (Fig. 2a), and calculated the relative frequencies of pSTAT3^+^ cells and that of the other four populations as well as Shannon diversity index (Fig. 2b, Supplemental Fig. S2A, B, and data not shown). Although pSTAT3^+^*JAK2*^amp^ and pSTAT3^–^*JAK2*^noamp^ cells were more common in metastases than in primary tumors, this relationship was not significant. To investigate the immune microenvironment, we then analyzed the frequencies of CD8^+^ T cells and the activated granzyme B^+^ subset of CD8^+^ T cells in each of the tumor samples using multicolor immunofluorescence (Fig. 2c). We found that metastatic lesions had significantly fewer infiltrating CD8^+^ T cells (Fig. 2d) and that a lower fraction of these were activated GZMB^+^CD8^+^ T cells (Fig. 2e, Supplemental Fig. S2C). Interestingly, CD8^+^ T-cell infiltration positively correlated with the frequency of pSTAT3^+^*JAK2*^noamp^ cells in primary tumors, whereas an inverse trend was observed in metastases (Fig. 2f). Conversely, CD8^+^ T-cell infiltration significantly correlated with pSTAT3^–^JAK2^noamp^ cell abundances in metastases, while an inverse trend was observed in primary tumors. Last, we determined that CD8^+^ T-cell infiltration inversely correlated with the Shannon index in both primary tumors and metastases (Fig. 2g).

### JAK/STAT pathway and drug effect correlative analyses

To investigate on-target activity of ruxolitinib, we investigated tissue-based and transcriptional evidence of JAK–STAT signaling inhibition comparing metastatic biopsies obtained pre-treatment and prior to cycle 2. A total of 14 patients underwent study biopsy with nine patients having baseline biopsy only, one patient having cycle 2 biopsy only, and three patients with both baseline and cycle 2 biopsies. Eleven of 13 baseline and all 4 metastatic biopsies passed tumor content quality control and were included in transcriptional analyses. In three patients with paired biopsies, there was a significant reduction in the fraction of pSTAT3^+^ cells after one cycle of treatment (mean pSTAT3^+^ 64.8% pre-treatment vs. 34.7% prior to cycle 2, *p* = 0.029; Fig. 3a). In addition, we evaluated whole- transcriptome RNA sequencing from biopsies obtained pre-treatment (*n* = 14) and prior to cycle 2 (*n* = 4). JAK/STAT-induced target genes *SOCS3* and *EGFR* were significantly repressed with ruxolitinib treatment (Student’s *t*-test *p* = 0.004 and *p* = 0.036, respectively) while JAK/STAT-repressed gene *LCK* was significantly upregulated (*p* = 0.007; Fig. 3b). As a more robust evaluation of pathway activity, we investigated two independently derived STAT3 signatures,^18,19^ both of which were decreased in cycle 2 biopsy samples (*p* = 0.041 and *p* = 0.065, respectively; Fig. 3c). As an unbiased approach, we identified differentially expressed genes using DESeq2 (ref. ^20^) performed pathway analysis of 44 genes with *p* < 0.001. The top two pathways were ‘‘Immune Response/OncostatinM signaling via JAK–STAT’’ in mouse and human cells (Fig. 3d). As an alternative pharmacocynamic measure, we also explored trends in IL-6 and CRP levels at baseline, cycle 2, and end of treatment; however, there were no convincing trends, though the power to detect differences was limited due to small patient numbers (Supplemental Table S2).

**Fig. 4:**
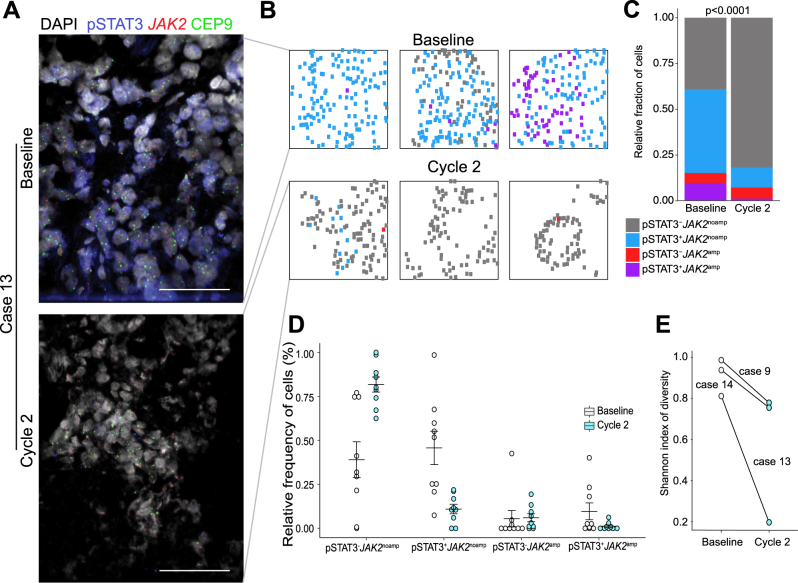
Immuno-FISH analysis of *JAK2* copy number and phospho-STAT3. **a** Representative images of iFISH for pSTAT3 and *JAK2* copy number at baseline and after cycle 2 in case 13. Scale bar 50  mm. **b** Maps showing topologic differences in the distribution of genetically and phenotypically distinct tumor cells based on their copy number signals for *JAK2* and pSTAT3 in three different regions of the tumor. **c** Summary of cell type frequencies in all patients at baseline and after cycle 2. The graph depicts the mean percentage of each cell type in all areas and the samples are combined. *p*  < 0.0001 (Chi-square test). **d** Dot plot depicting the relative frequencies of the four different cell types at baseline and at cycle 2. The significance of the difference between baseline and cycle 2 samples was calculated using Wilcoxon rank-sum test. Error bars, S.E.M. **e** Shannon index of cellular diversity in matched baseline and cycle 2 samples for *n*  = 3 pairs

To assess *JAK2* amplification and pSTAT3 status and impact of treatment at the single-cell level, we evaluated the four distinct populations via iFISH-positivity for pSTAT3 (i.e., pSTAT3^+^ and pSTAT3^-^) and JAK2 amplification (i.e., *JAK2*^amp^ and *JAK2*^noamp^; Fig. 4a, b, Supplemental Fig. S2A). The majority of pSTAT3^+^ cells were JAK2^noamp^, although *JAK2*-amplified cells were more likely to be pSTAT3^+^ (Fig. 4c). There was a decrease in pSTAT3^+^ tumor cells (*JAK2* amplified and non-amplified) in biopsies after two cycles of ruxolitinib treatment, confirming on-target activity of the drug even in the presence of *JAK2* amplification (Fig. 4c, d). We calculated Shannon diversity index of intratumor cellular diversity, a quantitative measure that reflects the number and amount of distinct phenotypes within a tumor, and detected a dramatic decrease in diversity in one patient and a less significant decrease in the other two (Fig. 4e). Collectively, these data demonstrate both tissue-based and transcriptional evidence of JAK–STAT pathway inhibition.

## Discussion

Ruxolitinib is FDA-approved for patients with intermediate or high-risk myelofibrosis and those with polycythemia vera who have had an inadequate response to or are intolerant of hydroxyurea based on results of several randomized phase III trials.^16,17^ Ruxolitinib is under active clinical investigation for a variety of other hematologic and oncologic indications. Based on preclinical evidence supporting the importance of the JAK2/STAT3 signaling pathway, we tested ruxolitinib, an oral JAK1/JAK2 inhibitor, in patients with metastatic TNBC and whose tumors demonstrated pSTAT3 expression. Disappointingly, we found no evidence of single-agent activity in this heavily pre-treated patient population, with no objective responses observed, and median PFS of only 1.2 months. The poor prognosis of this breast cancer subtype is exemplified by the median OS of only 4.5 months observed in this study. We observed expected hematologic toxicity; however, no major new toxicity signals were identified.

Given the lack of efficacy signal, we investigated the target effect of ruxolitinib. In transcriptional analyses of 11 pre-treatment and four cycle two tissue biopsy specimens, we identified suppression of JAK2-induced genes (*SOCS3, EGFR*) and STAT3 signatures, induction of JAK2-suppressed genes (including *LCK*), and JAK–STAT signaling as the top gene ontology in differential gene expression also consistent with a pharmacodynamic effect of ruxolitinib on tumor cells. In the three patients who had both pre-treatment and pre-cycle two biopsies, there was a significant and consistent decrease in the proportion of pSTAT3^+^ cells after ruxolitinib treatment with all demonstrating 40–55% relative decrease. Collectively, these observations suggest that ruxolitinib inhibits the target pathway within tumor tissue.

There are multiple potential hypotheses why anti-tumor activity was limited despite apparent on-target activity. It is possible that ruxolitinib leads to incomplete JAK–STAT inhibition or a cytostatic rather than a cytotoxic effect. Alternatively, we hypothesize that intratumoral heterogeneity could mediate resistance. Indeed, via iFISH analyses, we demonstrated a remarkable extent of intratumoral genomic and phenotypic heterogeneity within individual metastatic TNBC biopsies, despite enrollment criteria that required moderate-to-high pSTAT3 staining. Although there was significant decrease in pSTAT3^+^ cells, most patients had a substantial proportion of pSTAT3^–^ cells at baseline, suggesting that intratumoral heterogeneity could facilitate escape via expansion of resistant subclones.^21,22^

We explored the immune microenvironment given the importance of JAK2–STAT3 signaling in normal immune cells and the common co-amplification of *CD274*, encoding PD-L1 immune checkpoint inhibitor, with *JAK2* in triple-negative breast tumors.^23^ Strikingly, we found that metastases demonstrated fewer CD8^+^ and GZMB^+^CD8^+^ T cells than primary tumors, which may imply that the higher abundance of pSTAT3^+^ cancer cells may reflect a more active immune environment in primary tumors, and that these cells may directly recruit T cells as many chemokines and cytokines are direct targets of STAT3.^24^ Despite small numbers, greater intratumoral heterogeneity was associated with fewer infiltrating CD8^+^ T cells, suggesting that more diverse tumors may be less immunologically active. Previously, the *JAK2* mRNA level has been linked to an increased T-cell signature and improved survival in primary breast cancers.^25^ One hypothesis is that anti-tumor effects of ruxolitinib may have been balanced by inhibition of host anti-tumor immune response.

An important outcome of this study is characterization of the IL-6/JAK/STAT axis in metastatic TNBC. Overall, 40.4% of patients with available tissue to screen for this study demonstrated moderate- or high-levels of pSTAT3 by IHC. Although representing a selected population, these data suggest frequent activation in this pathway in advanced TNBC. Among enrolled patients who all had moderate- or high-pSTAT3, only a subset of these patients demonstrated gain or amplification of the *JAK2* locus, suggesting that mutations or alternate mechanisms of pathway activation may contribute to pathway activation.

Our study had several limitations. Although we did not demonstrate single-agent activity of ruxolitinib, we cannot rule out the possibility that ruxolitinib may add to the efficacy of chemotherapy or other targeted agents. Results of a non-randomized phase I study of ruxolitinib in combination with paclitaxel have recently been presented in abstract form, and clinical activity in patients with HER2-negative metastatic breast cancer has been observed.^26^ In preclinical models of IBC, ruxolitinib appears to add to the efficacy of taxanes (Peluffo et al., manuscript in preparation), and a prospective trial (TBCRC#039, NCT02876302) testing the combination ruxolitinib with paclitaxel in patients with triple-negative IBC is currently recruiting patients. In addition, only one patient with IBC (with TNBC) was enrolled so we cannot draw any conclusions about the efficacy of ruxotinib in IBC. To assess patient effects in addition to biologic effects, we incorporated patient-reported outcomes (PRO) in this study; however, the small number of participants and respondents emphasize the challenge of achieving adequate statistical power for PRO measures in early phase studies.

In summary, ruxolitinib as a single agent was well-tolerated overall but did not meet the primary efficacy endpoint in this treatment-refractory TNBC population. Correlative analyses demonstrate evidence of on-target activity, yet we identified multiple potential mechanisms of resistance including intratumoral heterogeneity with clonal escape and immune evasion.

## Patients and methods

### Patients

The study enrolled patients with histologically or cytologically confirmed invasive breast cancer with either metastatic disease or unresectable locally advanced disease (including IBC). Patients with IBC could have any combination of ER, PR, and HER2 status. All other patients had to have TNBC, defined as ER and PR <10% by immunohistochemistry (IHC) and HER2-negative (defined as IHC 0 or 1+ and/or FISH ratio <2.0). If there was a discrepancy between primary and metastatic biopsy, results of the metastatic biopsy were to take precedence for determination of eligibility. Other key eligibility criteria included at least one prior chemotherapy regimen in the metastatic setting and/or recurrence within 12 months of completion of (neo)adjuvant chemotherapy. Patients requiring chronic corticosteroids in excess of the equivalent of prednisone 10 mg daily were excluded, as were patients with active brain metastases. For the initial phase of the study reported here, only patients with pSTAT3 *T*-score of >5 were enrolled.

### pSTAT3 IHC

Archival, formalin-fixed, paraffin-embedded (FFPE) primary or metastatic tumor tissues were submitted for central pSTAT3 testing in the Department of Pathology at Brigham and Women’s Hospital using the following reagent: Cell Signaling, Phospho-STAT3 (Tyr705) (D3A7) XP Rabbit mAb (cat#9145L). IHC was performed on an automated instrument (Dako Autostainer Plus) according to prespecified protocols (Supplemental Fig. S3). A single pathologist (J.B.) reviewed all cases. A *T*-score was calculated based on percent stained cells and intensity of staining and interpreted as follows: >6, high-positive; 5, moderately positive; 3–4, weakly positive/equivocal; 0, negative.

### Protocol therapy, assessment schedule, and study endpoints

This was a non-randomized phase II study. The study was approved through Dana-Farber Cancer Institute Institutional Review Board (DFCI#12-024; NCT01562873) and informed consent was obtained from all subjects. Patients received ruxolitinib at a starting dose of 25 mg orally twice daily. Ruxolitinib dose modifications were per protocol according to a pre-defined algorithm. Up to three dose reductions were allowed (to 20 mg twice daily, 15 mg twice daily, and 10 mg twice daily). Complete blood counts were evaluated on days 1, 8, and 15 of the first cycle and on cycle day 1 thereafter. Physical examination, adverse event assessments, and chemistries were performed on days 1 and 15 of cycle 1 and on cycle day 1 thereafter. Adverse events were assessed according to the National Cancer Institute Common Terminology Criteria for Adverse Events (CTCAE) version 4.0. Tumor assessments occurred every two cycles. Confirmation of response >4 weeks later was required to deem a confirmed CR or PR. Blood samples for IL-6 and hs-CRP testing were collected at baseline, Cycle 2 Day 1, and off-treatment. Baseline tumor biopsies were required in patients with easily accessible or accessible disease. An on-treatment cycle 2 biopsy was required for patients with easily accessible (e.g., breast, chest wall, axillary node) disease and optional for other patients. An optional biopsy was to be performed at the time of disease progression. Patient-reported outcomes were assessed using the EORTC QLQ C-30 and MDASI on day 1 of cycles 1–3, and off-treatment.^27–30^ Patients were treated until disease progression, unacceptable toxicity, or withdrawal of consent.

The primary study endpoint was objective response rate by RECIST 1.1. Secondary endpoints included PFS, OS, toxicity profile, and clinical benefit rate (CR + PR + SD > 24 weeks).

### Statistical analysis

The study design is summarized in Fig. 1a. The study was designed to evaluate the activity of ruxolitinib in two cohorts: Cohort A, pSTAT3 *H*-score 5 or higher, and Cohort B, pSTAT3 *T*-score 3–4, and to distinguish between a response rate of 5% vs. 20% in each cohort separately. There was no plan for formal comparison of response rates between cohorts. Initially, patients were only to be entered into Cohort A. If at least two objective responses were observed within the first 21 patients enrolled in Cohort A, then Cohort A would proceed to full accrual and Cohort B would simultaneously open; otherwise, the study would close early. With this design, if at least five responses were observed in a total of 41 patients, the agent would be deemed worthy of future study (power for a true 20% response 0.90, type I error for a true 5% response 0.046). Only participants who received at least one dose of protocol therapy were included in the analysis.

### Genomic and transcriptomic analyses

Metastatic biopsies were obtained from patients with accessible disease prior to initiation of ruxolitinib (*n* = 13) and prior to initiation of cycle 2 in a subset of patients (*n* = 4). Two baseline metastatic biopsies with less than 20% tumor were excluded from subsequent analyses. For whole-genome and transcriptome analyses, total DNA and RNA were extracted. RNA sequencing was performed and read counts were converted to transcript per million values for individual gene, gene expression signature analyses.^31^

### Immunofluorescence

Immunofluorescence analyses were performed using 5 μm sections of FFPE tissues essentially as described.^32^ Briefly, slides were deparaffinized in xylene and hydrated in a series of descending ethanol. After heat-induced antigen retrieval in TRIS-EDTA (pH = 9) buffer, the samples were permeabilized with 0.5% TritonX-100, blocked with 5% goat serum in phosphate-buffered saline (PBS) and stained with anti-pSTAT3 antibody (Cell Signaling, cat#9131L) using a tyramide-based amplification kit (Perkin Elmer), followed by co-stain with anti-CD8 (ThermoFisher Scientific, cat#MA5-13473) and GranzymeB (Abcam, cat#ab4059) antibodies. Image analysis was performed on 3 × 3 montage images acquired by a Nikon Ti microscope attached to a Yokogawa spinning-disk confocal unit, ×40 Plan Apo objective, and OrcaER camera controlled by the Andor iQ software. pSTAT3 status was determined automatically using imageJ. CD8^+^ T cells were quantified automatically using ImageJ. GZMB^+^CD8^+^ T cells were quantified manually. Average counts of at least three montages per sample were quantified and used to calculate mean primary and metastases number of infiltrating CD8^+^ and percentage GZMB^+^CD8^+^ T cells.

### Immuno-FISH

Five-micrometer FFPE tissue sections were deparaffinized and rehydrated. Sections were digested with Proteinase K (1:1000 dilution) for 12 min at 37 ^o^C before proceeding to pSTAT3 staining, which was performed as described above. A FISH probe mix containing JAK2 BAC probe (RP11-927H16, labeled with SpectrumOrange dUTP by Nick Translation Kit; Abbott Molecular, according to the manufacturer’s recommendations) and CEP9 SpectrumGreen probe (Abbott Molecular) was then applied to the slides. Hybridization was performed for 7 min at 75 °C followed by overnight incubation at 37 °C in a humidified chamber. Next, the slides were washed in 0.4× SSC with 0.3% NP-40 for 2 min at room temperature, in 0.4× SSC with 0.3% NP-40 for 2 min at 74 °C, then in 2× SSC with 0.1% NP-40, in 2× SSC, and in PBS. After counterstaining with DAPI and mounting, slides were imaged using Nikon Ti microscope attached to a Yokogawa spinning-disk confocal unit, ×40 Plan Apo objective, and OrcaER camera controlled by the Andor iQ software. Five micromolar frozen sections were stained in the same manner, except they were first fixed with 4% PFA, and the digestion took place after the pSTAT3 staining. ImageJ was then used to analyze the images to automatically determine *x*–*y* coordinates, *CEP9* and *JAK2* copy number, as well as pSTAT3 expression status for each cell.

### Diversity, spatial, and statistical analyses for immunofluorescence and immuno-FISH data

To compute the diversity from the multicolor iFISH data, we categorized cells on each slide into the following four types based on their phosphorylation status of STAT3 and copy number of *JAK2*: pSTAT3^–^*JAK2*^noamp^, pSTAT3^+^*JAK2*^noamp^, pSTAT3^–^*JAK2*^amp^, pSTAT3^+^*JAK2*^amp^. Next, the fraction of each cell type on each slide was used to calculate the extent of intratumor diversity, which was measured by Shannon’s entropy; variance was similar between groups.^33^ We tested for significant differences in diversity between matched baseline and cycle 2 samples using the paired Wilcoxon rank-sum test, and also determined the differences in these fractions using the Wilcoxon rank-sum test. The R package Spatstat was used to visualize the topological distribution of each type in each slide. Finally, we evaluated the association between diversity and CD8^+^ T-cell counts for primary and metastatic samples using linear regression.

### Data availability

Tumor RNA sequencing data have been deposited into NCBI GEO, under accession code GSE107000.

## Electronic supplementary material

Supplemental Material(DOCX 1229 kb)
