# Supplementary material for: Phase II study of ruxolitinib, a selective JAK1/2 inhibitor, in patients with metastatic triple-negative breast cancer
Source: NPJ Breast Cancer. 2018 May 4;4:10. doi: 10.1038/s41523-018-0060-z (PMC5935675; doi:10.1038/s41523-018-0060-z)
Supplement: Supplementary file 1 — Supplemental Material(DOCX 1229 kb) [file 41523_2018_60_MOESM1_ESM.docx]

Phase II Study of Ruxolitinib, a Selective JAK1/2 Inhibitor, in Patients with Metastatic Triple-Negative Breast Cancer

Daniel G. Stover^1,2^*, Carlos R. Gil Del Alcazar^1^*, Jane Brock^1^, Hao Guo^3^, Beth Overmoyer^1^, Justin Balko^4^, Qiong Xu^3^, Aditya Bardia^5^, Sara M. Tolaney^1^, Rebecca Gelman^1^, Maxwell Lloyd^1^, Yu Wang^4^, Yaomin Xu^4^, Franziska Michor^3,7,8,9^, Vivian Wang^1^, Eric P. Winer^1^, Kornelia Polyak^1^, Nancy U. Lin^1^

^1^Department of Medical Oncology, Dana-Farber Cancer Institute, Boston, MA, USA

^2^Current affiliation: Department of Medical Oncology, Ohio State University Comprehensive Cancer Center, Columbus, OH, USA

^3^Department of Biostatistics and Computational Biology, Dana-Farber Cancer Institute, Boston, MA, USA

^4^Department of Biostatistics, Vanderbilt University Medical Center, Nashville, TN, USA

^5^Department of Cancer Biology, Vanderbilt University Medical Center, Nashville, TN, USA

^6^Department of Medical Oncology, Massachusetts General Hospital, Boston, MA, USA

^7^Department of Biostatistics, Harvard T. H. Chan School of Public Health, Boston, MA, USA ^8^Department of Stem Cell and Regenerative Biology, Harvard University, Cambridge, MA, USA

^9^Center for Cancer Evolution, Dana-Farber Cancer Institute, Boston, MA, USA

*These authors contributed equally to this work.

**Supplemental Table S1. Nadir blood counts on study.**

**Supplemental Table S2. IL-6 and hsCRP results.**

**Supplemental Figure S1. Time-to-event outcomes**

**Supplemental Figure S2. Individual sample pSTAT3 and JAK2 copy number immune-FISH**

**Supplemental Figure S3.** **Immunohistochemistry for pSTAT3 on paraffin embedded tissue**

**Supplemental Table S1. Nadir blood counts on study.**

| **Laboratory value** |  |
| --- | --- |
| WBC (median, range) | 4.1 (2.1-14.9) |
| Hct (median, range) | 29.5 (22-37.4) |
| Platelets (median, range) | 224 (105-481) |
| ANC (median, range) | 2380 (820-8560) |

**Supplemental Table S2. IL-6 and hsCRP results.**

|  | **C1D1** | **C2D1** | **Off-Treatment** |
| --- | --- | --- | --- |
| **# patients requiring assessment** | 21 | 12 | 21 |
| **# patients with IL-6 assessed** | 17 (81%) | 7 (58%) | 10 (48%) |
| **Median (range), pg/mL** | 12.5 (2.1-371.4) | 12.9 (0.8-212.5) | 20.5 (3.7-122.6) |
| **# patients with hsCRP assessed** | 20 (95%) | 9 (75%) | 12 (57%) |
| **Median (range), mg/L** | 7.0 (0.5-279.1) | 3.0 (0.1-72.7) | 57.6 (2.4-318.8) |

**Supplemental Figure S1. Time-to-event outcomes.** Kaplan-Meier curves of time-to-event outcomes. Median follow-up for survival was 4.5 months. **A.** Progression-free survival. Median PFS was 1.2 months (95% CI 0.97-1.84). **B.** Overall survival. Median OS was 4.5 months (95% CI 2.9-10.2).

**Supplemental Figure S2. Individual sample pSTAT3 and JAK2 copy umber immune-FISH.** **A.** Proportion of pSTAT3^+^ cells with *JAK2* copy number amplification or no amplification (cut off is 2.2 times the signal of CEP9). **B-C.** Quantification of immunofluorescence analysis depicting percentage of GZMB^+^CD8^+^ T cells (C) and fraction of pSTAT3^+^ cells (B). Error bars represent s.d. NLN: neck lymph node, PE: pleural effusion, RE: re-excision, RU: right upper, LU: left upper, LL: left lower.

**Supplemental Figure S3.** **Immunohistochemistry for pSTAT3 on paraffin embedded tissue.** Specimen fixation time is at least 6 hours for all cases. Four μm thick sections are baked at 37°C overnight, then deparaffinized and rehydrated (100% xylene x 4 for 3 minutes each, 100% ethanol x 4 for 3 minutes each, and running water for 5 minutes). Endogenous peroxidase activity is blocked with 3% hydrogen peroxide in methanol for 10 minutes and washed under running water for 5 minutes. Heat induced epitope retrieval is performed in EDTA buffer (pH 8.0) with a pressure cooker (Biocare Medical) at 122°C, to between 14 -17 PSI with the cycle lasting on average 45 minutes and cool down period approximately 20 minutes. IHC is performed on an automated instrument (Dako Autostainer Plus). A range of titers is tested for antibody and titer is calibrated using positive control staining, and negative staining of known (mouse) control tissue. Primary antibody (Cell Signaling, Phospho-Stat3 (Tyr705) (D3A7) XP® Rabbit mAb cat# 9145L) is diluted 1:200 and incubated overnight at 4^o^C, followed by detection with the Envision plus system (Dako) for 30 minutes in a humid chamber at room temperature. Sections are developed using 3,3’-diaminobenzidine (DAB) (Sigma, St. Louis, MO) as substrate and counterstained with Mayer’s hematoxylin. **A.** pStat3 scoring system. Scores for % stained cells and intensity of stains are summed and interpreted as listed. **B.** Representative images illustrating T-scores of 0, 3, 6, and positive control.
